# Scintigraphic Assessment of Pulmonary Flow in Patients After Pneumonectomy

**DOI:** 10.3390/diagnostics15060747

**Published:** 2025-03-17

**Authors:** Bogumił Maciąg, Małgorzata Edyta Wojtyś, Arkadiusz Waloryszak, Norbert Wójcik, Jarosław Pieróg, Krzysztof Safranow, Tadeusz Sulikowski, Tomasz Grodzki, Janusz Wójcik

**Affiliations:** 1 Department of Thoracic Surgery and Transplantation, Pomeranian Medical University in Szczecin, Alfreda Sokołowskiego 11, 70-891 Szczecin, Poland; 2 Clinical Department of Lung Diseases, Pomeranian Medical University in Szczecin, Alfreda Sokołowskiego 11, 70-891 Szczecin, Poland; 3 Department of Biochemistry and Medical Chemistry, Pomeranian Medical University, 70-111 Szczecin, Poland; 4 Clinic of General, Minimally Invasive and Gastroenterological Surgery, Pomeranian Medical University in Szczecin, 71-252 Szczecin, Poland

**Keywords:** pneumonectomy, pulmonary perfusion, pleural empyema, scintigraphy

## Abstract

**Background:** Pulmonary circulation typically shows flow divided between the right and left lungs, with a marked predominance of the right lung. Pneumonectomy reduces pulmonary circulation by ~50%, irreversibly changing the pulmonary perfusion characteristics. Here we assessed pulmonary flow after pneumonectomy and investigated how selected factors influenced pulmonary perfusion in this patient group. **Methods:** This study included 31 patients who underwent pneumonectomy complicated by postpneumonectomy pleural empyema, which was successfully treated, with long-term survival. Testing was conducted at a median of 1100 days after pneumonectomy, after flow stabilization. The control group comprised 31 subjects without pulmonary pathology. Pulmonary flow was assessed by scintigraphy using Technetium (99m-Tc). **Results:** The average single lung perfusion after pneumonectomy corresponded to the total perfusion in both lungs in the control group without statistic difference between comparable parameters (upper field, 21.35 vs. 22.129, *p* = 0.4; middle field, 47.15 vs. 49.62, *p* = 0.099; lower field 30.71 vs. 28.29, *p* = 0.14). Compared to those with left-sided pneumonectomy, patients with right-sided pneumonectomy exhibited increased upper field perfusion (22.61 vs. 19.82, *p* = 0.049) and decreased perfusion in the lower field (30.81 vs. 26.22, *p* = 0.049) and the combined middle and lower field (79.63 vs. 76.49, *p* = 0.046). Pulmonary flow was not significantly related to age, side of surgery, or empyema duration. **Conclusions:** Flow rate in the remaining lung after pneumonectomy corresponded to the total flow in both lungs in healthy controls. The perfusion ratio differed after right-sided versus left-sided pneumonectomy, which may be related to the initial anatomical differences of the right and left lung.

## 1. Introduction

Pulmonary respiratory function relies on the efficiency of a complex multifactorial process that includes pulmonary perfusion [1,2]. Analysis of pulmonary perfusion involves assessment of the distribution of perfusion to a single lung or both lungs and the degree of flow in the upper, middle, and lower lung fields, the intensity of which can be evaluated by isotope studies in plane and SPECT projections or even in the SPECT/CT version in pre- and postoperative evaluation [3,4,5,6,7,8]. In the present time, isotope studies are the most popular and have replaced angiography and digital subtraction angiography (DSA) of the pulmonary artery as well. While angio/CT is most useful in the assessment of acute perfusion disorders such as embolism, scintigraphy is the most effective method in quantitative assessment of flow in the peripheral vessels of the lung fields [9,10,11].

In physiology, the anatomical advantage of the right lung compared to the left lung (three lobes vs. two lobes) means a larger volume of the right lung compared to the left lung. The difference in size means that the flow norms for the right lung are in the range of 50–55% and for the left lung are in the range of 45–50% of the total pulmonary flow. The vertical body position and the influence of gravity cause the perfusion in the basal parts of the lungs to be nine times greater than in the apical parts of the lungs, causing a large difference in the flow between these two areas. The importance of upper field perfusion assessed by scintigraphy for a two-lung system and its impact on pulmonary function has been described and statistically confirmed. In this evaluation, flow ≤10% was the most effective, while >20% with the most limitation [1,2,8,11,12,13,14,15,16].

Pneumonectomy is the procedure involving the largest extent of pulmonary resection, resulting in approximately a 50% reduction of the pulmonary circulation vessels and respiratory surface area. This leads to irreversible changes in pulmonary perfusion characteristics of a remaining lung, although its detailed characteristics are not well documented in the literature. Our initial results from the pilot program showed an increase in flow intensity in the active areas and the use of flow reserves in the remaining fields, with particular emphasis on the increase in flow in the upper field of the left lung in relation to patients with the remaining right lung [17,18,19,20].

These changes are particularly important in cases with a potentially long follow-up period, due to the distant consequences of changes in the perfusion of a single lung. For a long time, pneumonectomy has been performed in extremely advanced, operable cases of lung cancer and other malignant or potentially malignant tumors of the respiratory system, with a significantly increased rate of complications, deaths, and failures. Over 40 years ago, a beneficial effect of postoperative pleural empyema on the length of survival was described in a group of patients who underwent surgery for lung cancer. Such a relation was observed in the available group of patients after pneumonectomy complicated by the development of postoperative pleural empyema (PPE), enabling the gathering of a study group [21,22,23,24,25,26].

In the present study, we aimed to evaluate the detailed pulmonary flow of a single lung by scintigraphy using Technetium (99m-Tc). We conducted it in an available group of patients after pneumonectomy that was complicated by the development of an empyema (PPE), due to long duration of follow up, which has not been widely analyzed so far. This allowed us to assess their pulmonary flow in the stabilized phase of complete cure and recovery after surgery. We paid particular attention to upper field perfusion as a potential indicator of the flow efficiency of the lung, similar to what previous studies have shown. Furthermore, we assessed the influence of selected factors—including age, side of surgery, and empyema duration—on the quality of pulmonary flow, examining whether there are additional factors that can have an impact on predicting patient outcomes after surgery. Our findings highlight the degree of single-lung flow reserve utilization, which is important in the context of planned therapy and management strategies for exacerbations of cardiopulmonary failure. Due to the limited number of studies on lung perfusion after pneumonectomy, our results may provide a basis for further exploration of this issue and the development of management strategies for patients with a single lung after surgery.

## 2. Materials and Methods

The study group comprised 31 patients who underwent pneumonectomy complicated by pleural empyema development. Pneumonectomy was performed as part of oncological treatment for 29 patients, while the remaining 2 patients underwent the procedure for non-oncological reasons. With regards to PPE treatment, 27 patients received accelerated treatment (AT) by accelerated repetitive lavage, 1 patient was treated by a thoracostomy technique with myoplasty of the empyema chamber, and 3 patients were treated with permanent pleural drainage. Within the whole group, the average duration of PPE treatment was 959 days (Table 1 and Table 2) [24].

Pulmonary perfusion was additionally assessed in the study group, because the group was controlled in terms of the effectiveness of empyema treatment and overall survival, which turned out to be above standards and enabled the assessment to be carried out. The treatment status of the empyema did not affect the flow rate. There were no active bronchial fistulas or changes in the lung image changing pulmonary perfusion, and initial qualification for pneumonectomy was a criterion excluding patients with emphysema or fibrosis. Moreover, the anatomy of the chest on the side opposite to the operated side was unchanged, although each patient had typical features of autothoracoplasty on the operated side.

This study also included a control group comprising 31 patients with two lungs, without pulmonary pathology, whose pulmonary perfusion results reflected flow standards in the literature. It was assumed that deviations of up to 2.5% of the flow values through an assessed lung were within the physiological picture. The control group was selected using propensity score matching for age and sex, according to the 1:1 rule, and radius matching (up to a 10-year age difference). The study and control groups did not significantly differ in terms of demographics, height, weight, BMI, age, and ejection fraction (Table 3).

Pulmonary perfusion was assessed using isotopic techniques with typical plane projections. Technetium-labeled human macroalbumin (99m-Tc) was intravenously administered 10 min before the examination, at a dose of 130 Bq, maintaining the vertical position of the chest. The scintigraphy image showed the distribution of radioactivity during the transient immobilization of isotope-labeled microparticles with a diameter of 15–100 μm in the capillary system of the pulmonary circulation [19]. The test result enabled the assessment of the intensity of use of circulatory vessels in the lung, although it did not allow for the evaluation of the flow rate in l/min.

**Figure 1:**
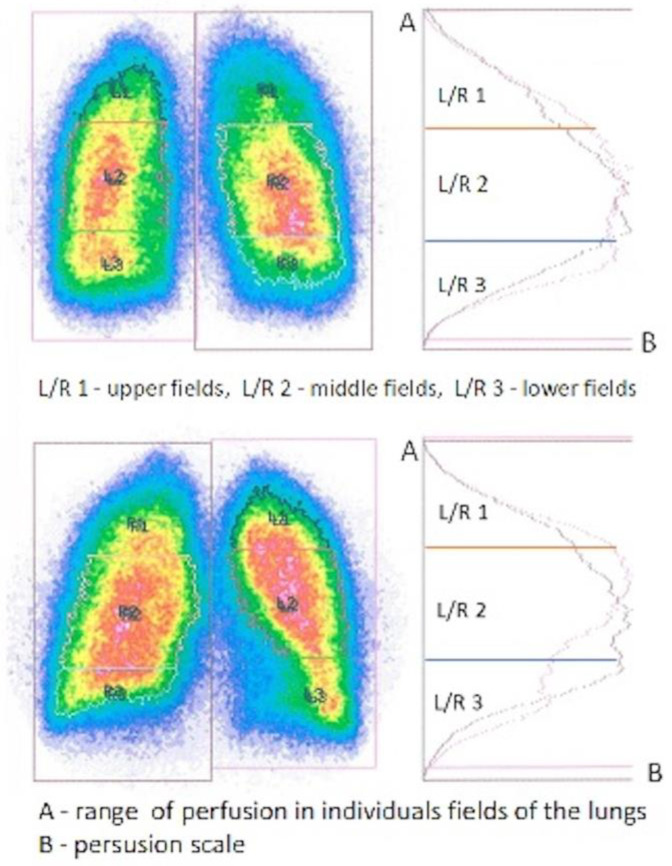
Original model scintigraphic view of both lungs divided into upper, middle, and lower fields with color variations of the perfusion intensity (red-highest intensity, blue-lowest intensity, green and yellow-moderate intensity of perfusion level) confirmed by isotope accumulation intensity curve plots.

**Figure 2:**
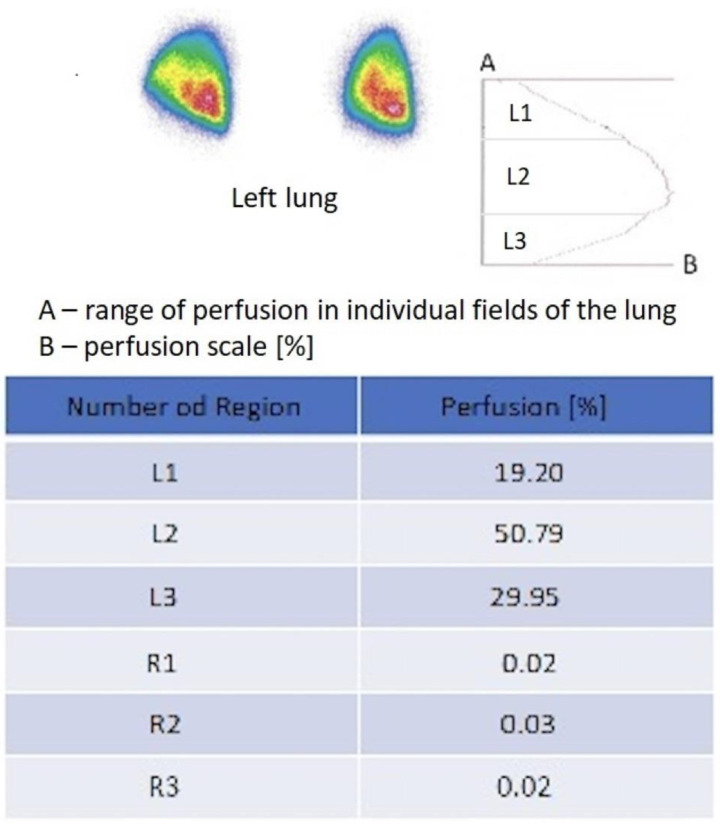
Original model scintigraphic report of left lung after right pneumonectomy. The perfusion map [%] shows scale of perfusion of 3 areas of the left lung: L1-upper, L2-middle, and L3-lower field and lack perfusion of the right lung (R1, R2, R3). The report is completed by isotope accumulation intensity curve plot. Red color indicates the area with the highest flow intensity, while blue indicates the area with the lowest flow. Yellow and green indicate areas corresponding to intermediate flow values.

Readings were obtained using a MB 9200 Gammacamera (Gamma Muvex) with NMS software and a Nucline AP Gammacamera with InterViewXP software (Mediso, Medical Imaging Systems). Results from both systems were standardized. Perfusion of a single lung was assessed in the study group, and flow through both lungs was assessed in the control group. Perfusion was assessed in three evenly spaced zones, each representing 1/3 of the height of the lung field. In the control group, it was assumed that, physiologically, 50–55% of the total pulmonary flow went to the right lung, and 45–50% to the left lung (Figure 1 and Figure 2). In the study group, perfusion assessment was performed a substantial time after pneumonectomy, during the stabilized flow phase. The shortest period from pneumonectomy to scintigraphic examination was 141 days, and the average/median interval for the entire study group was 1925/1100 days.

Statistical analyses included the Shapiro–Wilk test to examine normality of the data. Student’s *t*-test was used to analyze differences between the study group and the control group. Pearson’s linear correlation coefficient was determined to measure the strength of correlations between parameters within the study group. The threshold for statistical significance was *p* < 0.05.

## 3. Results

**Figure 3:**
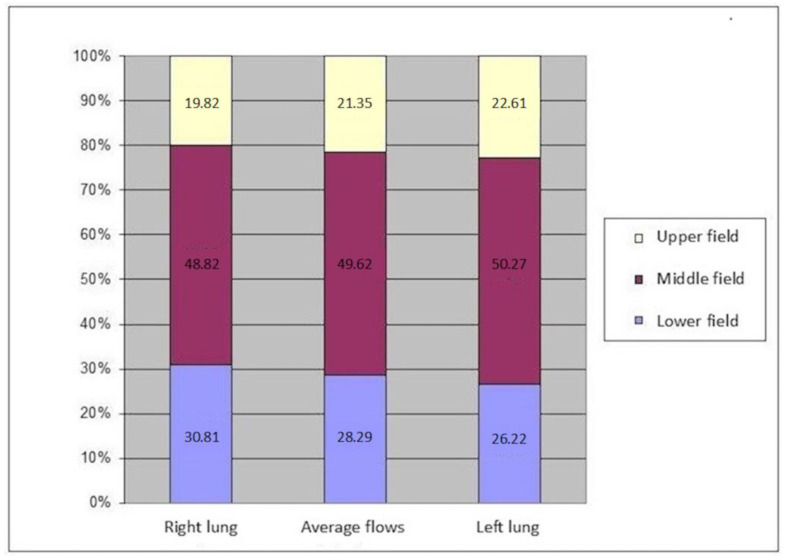
Average flow value in individual lung fields in the right lung (17/31), in the entire study group (31), and in the left lung (14/31).

The total physiological value of pulmonary flow at baseline was defined as 100%. Among patients in the study group, total pulmonary flow referred to the flow in the single remaining lung. We determined this flow value, as well as the flow values in each of the three lung fields. The study group was divided into patients operated on the left versus right sides. Table 4, Table 5, Table 6 and Table 7 and Figure 3 present the total flow values and flow values in individual lung fields in the patients after pneumonectomy.

**Figure 4:**
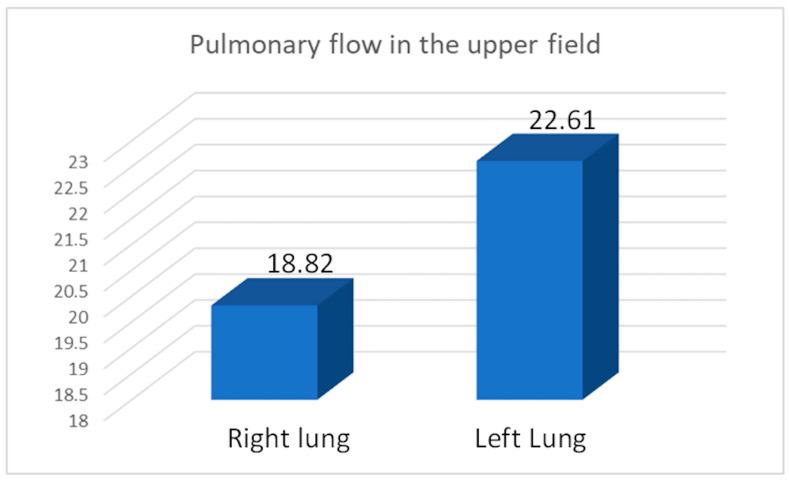
Average flow in the upper field (apical perfusion fraction AP% ratio) in the right (17/31) and left lung (14/31). Average flow in upper field in the entire study group after pneumonectomy (31) was 21.35.

We calculated the apical perfusion fraction (AP%), representing the percentage of perfusion in the upper field relative to the total perfusion of the assessed lung. The average AP% was 19.82 for the right lung, 22.61 for the left lung, and 21.35 for the entire study group after pneumonectomy (Figure 4). We compared the average flow rate values between the study and control groups (Table 8, Table 9, Table 10 and Table 11).

**Figure 5:**
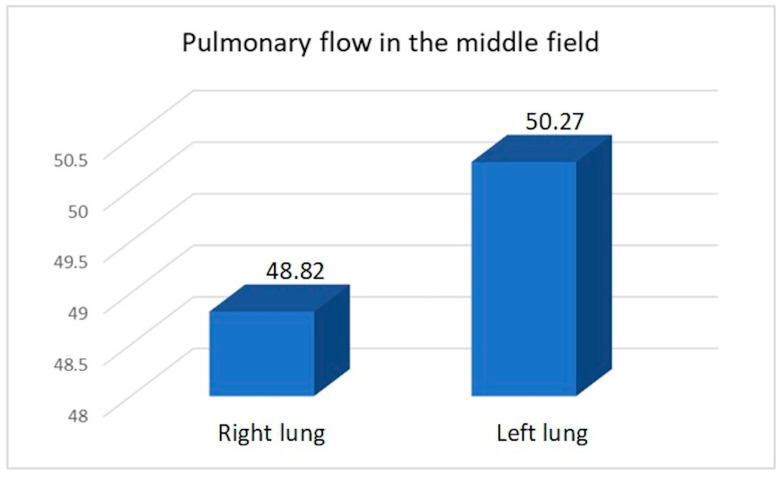
Average pulmonary flow in the middle field.

**Figure 6:**
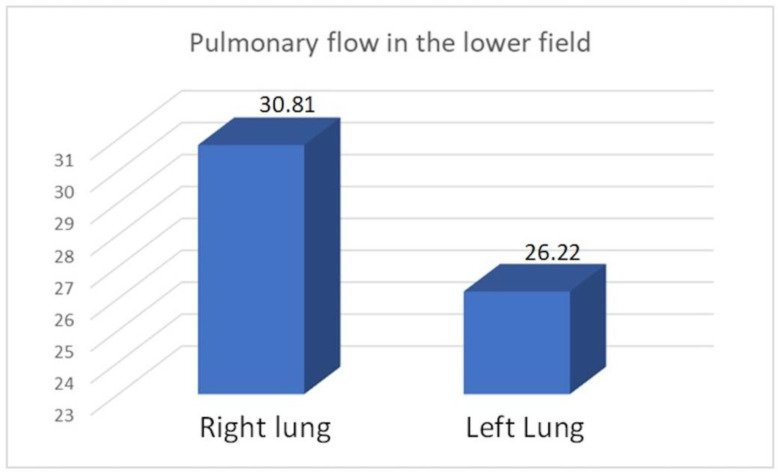
Average pulmonary flow in the lower field.

**Figure 7:**
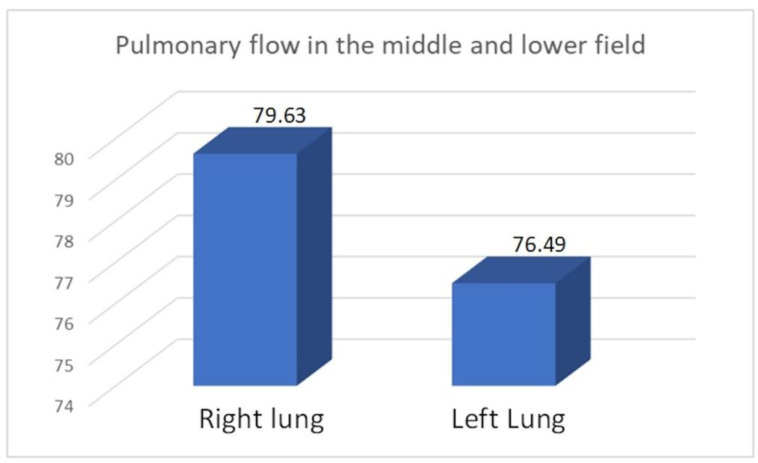
Average pulmonary flow in the combined middle and lower field.

We compared the perfusion values in the study group with the consolidated perfusion values of both lungs in the control group and found no significant difference in the upper field (21.35/22.13, *p* = 0.40), middle field (49.62/47.15, *p* = 0.099), or lower field (28.29/30.71, *p* = 0.14). The flow of the combined middle and lower fields was essentially the same in both groups: 77.91 vs. 77.85. Similarly, we found no significant differences between the consolidated upper field flows in both lungs in the control group compared to the upper field perfusion value for the right pneumonectomy group (22.61/22.57, *p* = 0.978) or the left pneumonectomy group (19.82/21.59, *p* = 0.125). Within the study group, we compared perfusion values between patients with a remaining right versus left lung (Table 12, Table 13, Table 14 and Table 15) (Figure 5, Figure 6 and Figure 7).

We observed increased upper field perfusion in the remaining left lung after right-sided pneumonectomy compared to the remaining right lung after left-sided pneumonectomy (22.61 vs. 19.82, *p* = 0.049). On the other hand, compared to patients who underwent right-sided pneumonectomy, those who underwent left-sided pneumonectomy exhibited increased perfusion in the lower field (30.81 vs. 26.22, *p* = 0.049) and in the combined middle and lower field (79.63 vs. 76.49, *p* = 0.046).

There was no statistical relation between the perfusion of the upper field and the age of patients or time interval since pneumonectomy in the study group (Table 16 and Table 17).

Pearson’s linear correlation (R) analysis showed that the upper field perfusion results were not related to age (R = −0.23465, *p* = 0.20) nor time interval since pneumonectomy (R = 0.078226, *p* = 0.67).

## 4. Discussion

In the current analysis, we examined the efficiency and effectiveness of pulmonary circulation in patients after pneumonectomy and in controls with two normal lungs. The pulmonary circulation is a low-pressure, low-resistance vascular system characterized by the ability to adapt to increased flow without a significant increase in pulmonary artery pressure (averaging 15 mmHg). This is possible due to the activation of additional arteriovenous connections in the capillary area [1,2,11,13,16]. Another mechanism of pressure stabilization in the pulmonary circulation, when there are flow restrictions in a lesioned lung, is to increase the flow in the other lung. The degree of flow through specific areas of the lung may also be regulated via a reflex pathway that depends on the degree of blood oxygenation and ventilation [1,2,11,13,16].

One of the main mechanisms for stabilizing pulmonary pressure is through the physiological flow deficit in the upper lung fields, which provides a flow reserve for an increase in flow dynamics in response to increased demand of the respiratory process or increased flow resistance. Under conditions of optimal efficiency and large flow reserves, the perfusion level of the upper field for each lung does not exceed (or only slightly exceeds) 10% of the total pulmonary flow [1,2,16,17,19], as was confirmed in our control group. The physiological flow deficit in the upper fields, relative to the rest of the lungs, also accounts for the overall proportion of flow in the pulmonary circulation in an upright position, based on gravitational forces, and affects flow through both a single lung and through the two-lung system [1,2,17,18,19,20]. This principle was confirmed by our analysis of the results in the test and control groups. Indeed, the upper lung field flow in the pneumonectomy group did not significantly differ from the pooled flow in the upper fields of both lungs in the control group (*p* = 0.40).

Pneumonectomy eliminates half the volume of the pulmonary circulatory system, forcing the greatest use of flow reserves in the remaining lung. The expected end result is an increase—and in some cases even a doubling of the flow values in the evaluated fields—due to displacement of the cardiac output volume to a 50% reduced pulmonary circulation volume (Table 8, Table 9, Table 10 and Table 11) [17,18,20]. The results in our study group seem to confirm this principle.

Our present findings showed that the flow reserves were reduced in the upper field of the single remaining lung in our study group. After pneumonectomy, none of the patients exhibited an upper field flow of 10% or lower. In the study group, the upper field flow was between 10–20% (average, 17.57) in 13 patients (41.93%), and it was above 20% (mean, 23.99) in 18 patients (58.06%). The mean flow value in the upper field in the total study group was 21.35%, which was similar to the average flow value of 22.13% in the upper fields of both lungs in the control group. It can be assumed that the group with a flow rate in the range of 10–20% (41.93% of patients) had a baseline perfusion of the upper fields of <10% and had the largest flow reserves of both lungs.

The principle of the flow values of a selected field in the study group being similar to the total flow value of equivalent fields of both lungs in the control group was evident in our comparison of the mean flow values of the middle fields (49.62% vs. 47.15%, *p* = 0.099), lower fields (28.29% vs. 30.71%, *p* = 0.14), and the sum of the lower and middle fields (77.91% vs. 77.85%). These observations indicate that the upper limit of perfusion values in the upper field in both the control group (for both lungs combined) and the group after pneumonectomy (for the one remaining lung) does not exceed the limit of 28–29% of flow values. The majority (58.06%) of patients in the study group exhibited submaximal (12 patients: 21–24.92%) or maximal (6 patients: 25.44–28.5%) utilization of flow reserves. There was an advantage of 14 vs. 4 combined submaximal and maximal increased upper pulmonary flows, as well as 4 vs. 2 maximal increased upper pulmonary flows in comparison of the left to the right lung in the study group.

Moreover, we observed a statistically significant increase in the perfusion of the upper field of a remaining left lung (after right-sided pneumonectomy) compared to the remaining right lung (after left-sided pneumonectomy) (22.61 vs. 19.82, *p* = 0.04). This is due to the volume advantage of the right lung over the left lung and the increased flow area in the combined middle and lower fields (79.63 vs. 76.49, *p* = 0.046). Our results did not show that flow volume in the upper field was significantly associated with patient age (*p* = 0.20) or with the time interval since pneumonectomy (*p* = 0.67).

Flow coefficient values—including the upper field flow coefficient value (AP%)—can be used to standardize perfusion analyses of individual lung fields. This value should be about 10% under physiological conditions and approaches the upper flow limit after exceeding 25%. We found a significant difference between the left and right remaining lungs of the study group (22.62 vs. 19.82, *p* = 0.04). The results are highly important for patients with potentially long post-pneumonectomy survival, such as non-oncologic patients and oncologic patients with a good prognosis. The 5-year and 10-year survival results in our study group were better than expected. Our observations highlight the degree of utilization of the flow reserves of a single lung, which is significant in the context of scheduled therapy and management strategies for exacerbations of cardiopulmonary failure.

The results identify patients with higher risk due to a high level of flow in the upper field and a high AP% value, with likely a particular focus on patients who undergo right-sided pneumonectomy, although the low number of patients is the main limitation of this study.

In conclusion, our present findings showed that the amount of flow in a single lung after pneumonectomy closely corresponded to the sum of the flow in both lungs in healthy subjects. Our results did not confirm that the degree of pulmonary flow was significantly related to age or time interval from pneumonectomy. We found that flow significantly differed between the right and left remaining lungs after pneumonectomy, in terms of perfusion of the upper, lower, and combined middle and lower fields, which may be related to the initial anatomical differences between the right and left lung, and this requires further research.

## Figures and Tables

**Table 1:** Characteristics of the study group.

Number of patients	31
Males	27
Females	4
Age, range/average/standard deviation in years	47–75/61/9
Right operated side	17
Left operated side	14
Time period for pneumonectomy performance	1995–2015
Operated for oncological reasons	29
Operated for non-oncological reasons	2
Empyema treatment using accelerated treatment (AT)	27
Empyema treatment with pleural drainage	3
Empyema treatment by thoracostomy and myoplasty	1
Duration of empyema treatment, range in days	34–16,469
Duration of empyema treatment, average/SD in days	959/2967
Interval from pneumonectomy to scintigraphy, range in days	141–16,469
Interval from pneumonectomy to scintigraphy, average/median/SD in days	1925/1100/3058

SD, standard deviation.

**Table 2:** Updated survival results of study group patients treated for lung cancer.

5-year overall survival	23/29 (79.3%)
10-year overall survival	19/29 (65.5%)
Follow-up period for oncology cases, range in months	16–181
Follow-up period for oncology cases, average/SD in days	1472/1478

SD, standard deviation.

**Table 3:** Characteristics of the study and control groups.

Comparative Feature	Study Group	Control Group
Number of patients	31	31
Male/Female	27/4	27/4
Age at pneumonectomy, range in years	47–75	37–75
Age at pneumonectomy, average/SD in years	61/8.5	61/9.9
Statistical significance of age difference	*p* = 0.40	
Height, range in cm	146–196	156–182
Height, mean/SD in cm	169/9	170/5
Statistical significance of height difference	*p* = 0.52	
Weight, range in kg	36–105	55–122
Weight, average/SD in kg	73.9/17.4	80.1/16.1
Statistical significance of weight difference	*p* = 0.16	
BMI, range	14.9–40.8	18.4–40.8
BMI, average/SD	25.6/5.4	27.8/6.2
Statistical significance of BMI difference	*p* = 0.16	
Left ventricular ejection fraction, range	33–70	33–80
Left ventricular ejection fraction, average/SD	52/9.6	56/10
Statistical significance of the difference in left ventricular ejection fraction	*p* = 0.10	

SD, standard deviation.

**Table 4:** Results of the upper field flow in the entire study group according to the assessed lung.

Nr	Side	AP%	Nr	Side	AP%
1.	right	27.68	17.	left	22.34
2.	right	24.92	18.	left	21
3.	right	19.85	19.	left	22.36
4.	right	16.59	20.	left	18.23
5.	right	17.82	21.	left	25.44
6.	right	21	22.	left	23.51
7.	right	17.81	23.	left	24.06
8.	right	14.86	24.	left	23.9
9.	right	18.04	25.	left	21.74
10.	right	15.47	26.	left	19.2
11.	right	19.32	27.	left	23.3
12.	right	26.1	28.	left	21.1
13.	right	18.42	29.	left	28.5
14.	right	19.6	30.	left	27.1
15.	left	13.22	31.	left	22.5
16.	left	27.17			

**Table 5:** Ranges of total flow and flow in individual lung fields in the whole study group including patients after right and left pneumonectomy.

Flow Range	Average Flow	SD	Minimum Flow	Maximum Flow
Whole lung	99.27	1.04	95.4	100
Upper field	21.35	3.96	13.22	28.5
Middle field	49.62	6.05	40	65.92
Lower field	28.29	6.52	11.04	38.3
Lower and middle field	77.91	4.39	68.9	86.69

SD, standard deviation.

**Table 6:** Flow of individual lung fields in the right lung in patients after left pneumonectomy.

Lung Fields	Average Flow	SD	Minimum Flow	Maximum Flow
Upper field	19.82	3.88	14.86	27.68
Middle field	48.82	6.32	40	62.32
Lower field	30.81	6.71	15.51	38.3
Lower and middle field	79.63	4.21	71.8	85.02

SD, standard deviation.

**Table 7:** Flow of individual lung fields in the left lung in patients after right pneumonectomy.

Lung Fields	Average Flow	SD	Minimum Flow	Maximum Flow
Upper field	22.61	3.66	13.22	28.5
Middle field	50.27	5.94	40.6	65.92
Lower field	26.22	5.76	11.04	33.5
Lower and middle field	76.49	4.13	68.9	86.69

SD, standard deviation.

**Table 8:** Comparison of average flow rates in the lung of the study group and the combined flow of both lungs in the control group – Upper Field Flow Rate/AP% and Middle and Lower Fields Flow Rate. Single unipolar lung flow rates of the control group were not compared.

Group Category	Upper Field Flow Rate/AP%	SD	Middle and Lower Fields Flow Rate	SD
Study group	21.35	3.96	77.91	4.39
Control group (unipolar lung)	11.43	2.11	38.99	4.14
Control group (both lungs)	22.13	3.71	77.85	3.72

SD, standard deviation; AP%, apical perfusion fraction.

**Table 9:** Comparison of average flow rates in the lung of the study group and the combined flow of both lungs in the control group – Middle Field Flow Rate and Lower Field Flow Rate. Single unipolar lung flow rates of the control group were not compared.

Group Category	Middle Field Flow Rate	SD	Lower Field Flow Rate	SD
Study group	49.6	6.05	28.29	6.52
Control group (unipolar lung)	23.59	3.05	15.41	4.17
Control group (both lungs)	47.15	4.99	30.71	6.07

SD, standard deviation.

**Table 10:** Comparison of average flow rates of the left lung between the control group and patients in the study group who received right-sided pneumonectomy. Single unipolar lung flow rates of the control group were not compared.

Group Category	Upper Field Flow Rate/AP%	SD	Middle and Lower Fields Flow Rate	SD
Group after right-sided pneumonectomy	22.61	3.66	76.49	4.13
Control group (unipolar lung)	11.67	2.36	35.96	2.38
Control group (both lungs)	22.57	4.13	77.4	4.14

SD, standard deviation; AP%, apical perfusion fraction.

**Table 11:** Comparison of the average flow rates of the right lung between the control group and patients in the study group who received left-sided pneumonectomy. Single unipolar lung flow rates of the control group were not compared.

Group Category	Upper Field Flow Rate/AP%	SD	Middle and Lower Fields Flow Rate	SD
Group after left-sided pneumonectomy	19.82	3.88	79.63	4.21
Control group (unipolar lung)	11.14	1.8	42.7	2.56
Control group (both lungs)	21.59	3.19	78.39	3.19

SD, standard deviation; AP%, apical perfusion fraction.

**Table 12:** Comparison of upper field flow in remaining right versus left lung after pneumonectomy in the study group.

Study Side—Upper Field Perfusion/AP%	Average Value	SD	Minimum Value	Maximum Value	*p* Value
Right lung	19.82	3.88	14.86	27.68	0.049
Left lung	22.61	3.66	13.22	28.5	

SD, standard deviation; AP%, apical perfusion fraction.

**Table 13:** Comparison of middle field flow in remaining right versus left lung after pneumonectomy in the study group.

Study Side—Middle Field Perfusion	Average Value	SD	Minimum Value	Maximum Value	*p* Value
Right lung	48.82	6.32	40.0	62.32	0.51
Left lung	50.27	5.94	40.6	65.92	

SD, standard deviation.

**Table 14:** Comparison of flow in the lower field in remaining right versus left lung after pneumonectomy in the study group.

Test Side—Lower Field Perfusion	Average Value	SD	Minimum Value	Maximum Value	*p* Value
Right lung	30.81	6.71	15.51	38.3	0.049
Left lung	26.22	5.76	11.04	33.5	

SD, standard deviation.

**Table 15:** Comparison of flow in the lower and middle fields in remaining right versus left lung after pneumonectomy in the study group.

Test Side— Perfusion of the Middle and Lower Fields	Average Value	SD	Minimum Value	Maximum Value	*p* Value
Right lung	79.63	4.21	71.8	85.02	0.046
Left lung	76.49	4.13	68.9	86.69	

SD, standard deviation.

**Table 16:** Comparison of flow in the upper field depending on age.

Age Category (Number of Patients)	Average Value	SD	Minimum Value	Maximum Value	*p* Value
47–61 years (14)	22.72	2.72	18.23	28.5	0.20
62–75 years (17)	20.02	4.36	13.22	27.68	

**Table 17:** Comparison of flow in the upper field depending on the time since pneumonectomy.

Time Interval (Number of Patients)	Average Value	SD	Minimum Value	Maximum Value	*p* Value
141–1087 days (15)	20.87	3.9	13.22	27.1	0.67
1100–16,469 days (16)	21.79	4.08	14.86	28.5	

## Data Availability

Data available on request due to ethical reasons. Data on which the study was based can be found in the Polish National Registry of Transplantation; patient’s records are kept in the archives of the Department of Thoracic Surgery and Transplantation, Pomeranian Medical University, Szczecin, Poland.
